# Mycorrhizal fungi arbuscular in forage grasses cultivated in Cerrado soil

**DOI:** 10.1038/s41598-022-07088-5

**Published:** 2022-02-24

**Authors:** Leidiane dos Santos Lucas, Aurelio Rubio Neto, Jadson Belem de Moura, Rodrigo Fernandes de Souza, Maria Eduarda Fernandes Santos, Lorena Fernandes de Moura, Elitania Gomes Xavier, José Mateus dos Santos, Ryan Nehring, Sandro Dutra e Silva

**Affiliations:** 1 Graduate Studies in Agricultural Sciences / Agronomy, Instituto Federal Goiano, Rio Verde, Goiás Brazil; 2 Sedmo - Soil Research Group, Ecology and Dynamics of Organic Matter, Evangelical College of Goianésia, Goianesia, Goiás Brazil; 3 Graduate Studies in Natural Resources of the Cerrado, State University of Goiás, Anápolis, Goiás Brazil; 4 SEMPA Seeds Technology, Goiania, GO Brazil; 5 Graduate Studies in Social, Technological and Environment Science, Evangelical University of Goiás, Anápolis, Goiás Brazil; 6 Department of History and Philosophy of Science, University of Cambridge, Cambridge, UK

**Keywords:** Microbial ecology, Agroecology

## Abstract

The Cerrado is one of the most important regions for agricultural development in the world and is the main productive breadbasket of the Americas. One of the main agricultural activities in the region is high-tech livestock. Cerrado soils are predominantly low in fertility, and arbuscular mycorrhizal fungi play a fundamental role in plant nutrition in this biome. Understanding the behavior of mycorrhizal fungi in the soil under pasture is essential for the development of more efficient and sustainable management practices. Thus, this work aims to verify the activity of arbuscular mycorrhizal fungi in different species of forage grasses cultivated in cerrado soil. To measure mycorrhizal activity, soil spore density factors and mycorrhizal colonization rates in roots of 14 forage grass genotypes were investigated. No significant differences were identified in spore density values between the investigated genotypes. Panicum maximum cv and Mombasa showed the lowest values of mycorrhizal colonization, and the highest values were found in the roots of *Brachiaria decumbens*. Among the identified genera associated with the rhizosphere of the genotypes studied, *Gigaspora, Scutelospora* and *Sclerocysts* are less frequent, which indicates that the association with these fungal genera is less recurrent than with the others.

## Introduction

The Cerrado is the second largest Brazilian biome, extending over an area of 2,045,064 km^2^ and spanning eight states of Central Brazil: Minas Gerais, Goiás, Tocantins, Bahia, Maranhão, Mato Grosso, Mato Grosso do Sul, Piauí and Distrito Federal^1^. It is divided by three of the largest hydrographic basins in South America, with regular rainfall indices that provide great biodiversity. After the Amazon, the Cerrado today is considered the last agricultural frontier of the Americas^2–4^.

The Cerrado is considered to be a “biodiversity hotspot”, as it has one of the greatest levels of biodiversity on the planet. Such levels of biological diversity are achieved due to it being a transition biome that is in direct geographic contact with other important South American biomes, such as the Amazon, Caatinga, Atlantic Forest, Pantanal and Bolivian Chacos^2,5^. From a natural history perspective, the Cerrado could be considered a biogeographic region that is more than 40 million years old^6–9^. Such biogeographic continuity has resulted in a symbiosis between flora, fauna and microorganisms^3^. Due to its privileged location, the Cerrado stands out as one of the most important agricultural frontiers in the world^3,4^, with much of the area today consisting of large-scale industrial agriculture and degraded pastures^10,11^.

With the current productive paradigm, environmental sustainability is considered to be an important factor in determining the success of production systems. In agriculture, no-tillage systems are being promoted as a cultivation system that promotes soil and water conservation^12,13^. For no-tillage systems to maintain levels of productivity, vegetation cover is an adequate factor. For conditions in the Cerrado, vegetation cover must have a low carbon–nitrogen ratio, which decreases the decomposition speed and increases the time in which the cover protects the soil from erosive processes^14^. In this sense, grasses stand out as ideal plant cover for no-tillage systems in the Cerrado, in addition to being important forages for animal grazing^15^. Natural cover systems based on symbiotic systems may also reduce the use of herbicides, which have been used extensively for no-till systems in the tropical climates of Brazil^16^.

Generally, Cerrados are environments that naturally offer adverse abiotic conditions for plant growth and development. With low phosphorus levels and irregular rainfall, vegetation depends directly on the performance of mycorrhizal fungi to resist surviving such conditions, which is attributed to the association between fungi and plants as an important factor in building resilience to stressful situations^1,17–19^.

The association of mycorrhizal fungi with vegetation started its evolution in tropical regions, and there are even species that are found only in these regions^20^. Today, however, the presence of these fungi is reported in different regions of the planet, regardless of climate^19,21–25^.

The average density of mycorrhizal fungal species in the soils of the Cerrado varies from 25 to 50 spores per 50 cm^3^ of soil on average. In the neighboring region of the Caatinga, there is a variation in the number of propagules of these fungi, probably due to differences in the plant community, and in relation to chemical composition and land use, with ranges containing high phosphorus^6,26^.

For agricultural production, combined with the recovery of degraded areas, understanding the behavior of forage grass species with soil biology is essential for the development of more efficient practices for the management of natural resources. Cerrados are environments that offer adverse abiotic conditions for plant growth and development, with low levels of phosphorus and a limited water regime, and the development of their vegetation depends directly on the action of soil microorganisms. Under these conditions, mycorrhizal fungi stand out as organisms that promote plant growth and contribute to plant resilience to stressful situations^1,17–19,27^. Therefore, this work aims to verify the mycorrhizal population dynamics in forage grass species in Cerrado soils.

## Materials and methods

The experiment was conducted at the Agrostological Field of the Ricardo Fontoura Experimental Station of the Cerrado, which is part of the Evangelical College of Goianésia, in the state of Goiás, Brazil. The climate is classified as a tropical season (AW) characterized by two well-defined seasons: dry and rainy^14^. The density of spores and the mycorrhizal colonization rate of 14 varieties of forage grasses were evaluated (Table 1).

**Table 1:** Forage grasses installed at the Agrostological Field of the Ricardo Fontoura Experimental Station of the Cerrado, Evangelical College of Goianésia.

Forage Grasses
*Urochloa decumbens*
*Brachiaria Ruziziensis*
*Brachiaria brizantha cv marandu*
*Brachiaria brizantha cv piatã*
*Brachiaria brizantha cv. Xaraes*
*Brachiaria brizantha cv. Paiaguas*
*Brachiaria brizantha cv. Ipyporan*
*Brachiaria brizantha cv. Humidicola*
*Megathyrsus maximus cv. Mombasa*
*Megathyrsus maximus cv. Kenya*
*Megathyrsus maximus cv. Zuri*
*Megathyrsus maximus cv. Aruana*
*Megathyrsus maximus cv. Tamani*
*Megathyrsus maximus cv. Massai*

Samples of rhizospherical soil containing the treatment roots described in Table 1 were collected. Each sample taken to the laboratory was composed of 3 simple samples randomly collected from each plot. The design had a completely updated design with 6 replicates. Sampling was carried out at the end of the dry season in September 2020.

The analyses were carried out in the laboratory of agricultural microbiology of the Evangelical College of Goianésia. The spores of arbuscular mycorrhizal fungi (AMF) were extracted from 50 cm^3^ of rhizospherical soil by wet sieving^15^ followed by centrifugation in water and a 50% sucrose solution. The spores were separated according to their phenotypic characteristics, such as color, size and shape, composing the different morphotypes under stereoscopic binocular magnifying glass^28^.

To determine the percentage of colonization, the roots were clarified and ordered with 0.05% Trypan Blue in lactoglycerol^16^ and the colonization was evaluated under under a stereoscopic microscope, following the quadrant intersection technique^17^.

To identify the genera of AMF from morphological characteristics, the spores were separated according to their morphotypes and mounted on blades with pure polyvinyl-lactoglycerol (PVLG) and PVLG mixed with Melzer (1:1 v/v). To support the identification work, original articles from the descriptions of species were provided on the website of the "International Culture Collection of Arbuscular and Vesicular–Arbuscular Mycorrhizal Fungi"^18,29^.

The data were submitted to variance analysis by the Assistat^30^ analyses of canonical correspondence were performed by the Past^20,31^ software. Spore density variables, and the rate of mycorrhizal colonization was determined by a 5% Tukey test. The presence of identified genera was used as the parameter for multivariate analysis.

The conduct of the experiment followed the International guidelines of the IUCN Policy Statement on Research Involving Species at Risk of Extinction. The plant material studied, as a perennial species, is available for study and review at the agrostological field of the Evangelical College of Goianésia.

## Results and discussion

**Figure 1:**
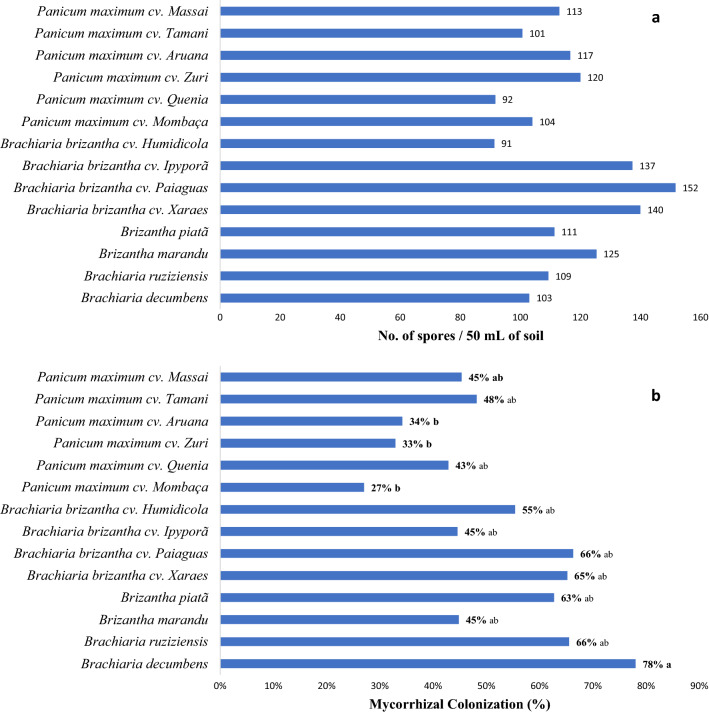
Density of mycorrhizal fungi spores in rhizospherical soil (**a**) and rate of mycorrhizal colonization (**b**) of different forage grasses in Cerrado soils.

For the determination of ecological interactions between AMF and forage grasses, the values of density of spores in the soil, mycorrhizal colonization rate in the root, and the presence of genera of AMF associated with the rhizosphere are used as parameters. No significant difference was verified between the analyzed varieties when investigating the density of spores in rhizospherical soil of forage grass varieties in Cerrado soils (Fig. 1a). P values for spore density were 0.2868 and for colonization were 0.1662.

The mycorrhizal colonization rate showed a significant difference (*p* < 0.05). Urochloa decumbens presented the highest mycorrhizal colonization rate (78%) compared to the others. The species Megathyrsus maximus cv. Zuri, Megathyrsus maximus cv. Aruana and Megathyrsus maximus cv. Mombasa presented the lowest values of mycorrhizal colonization in its roots, 33%, 34% and 27%, respectively (Fig. 1b).

The absence of a significant difference in spore density values is because the varieties were installed in the same area and where they are probably being colonized by the same fungal species, since they present low specificity. It is expected that there is no difference in the sporulation of fungi from the same area, since spore production is a response of the fungus and not of the host plant. The production of spores is a reflection of the fungus to environmental changes that, under stressful conditions, begin to produce spores as a resistance structure^21,22,32^.

The samplings were carried out at the end of the dry season. In the cerrado, the climate is classified as tropical seasonal (AW) characterized by two well-defined seasons: a dry period and a rainy period^33^. These climatic conditions are considered stressful for most soil organisms due to the absence of rainfall for more than 4 consecutive months, which explains the high values of spore density values in the soil^12,23^.

Forage plants do not present specificity for the colonization of mycorrhizal fungi and can be colonized by more than one species of fungus^24^. However, some plant species have higher mycorrhizal colonization rates than others. Different species may present different values of colonization in the same environment, which is a reflection of the evolutionary adaptability of this symbiotic association^34–37^.

Mycorrhizal colonization values indicate the intensity to which fungi have to associate with vegetation to assist with functions such as water and nutrient absorption (MOREIRA; SIQUEIRA, 2006). Because it is the same soil, the variation in mycorrhizal colonization values is explained by the physiological differences of plants and not fungi. This behavior can be observed when comparing forage plants, such as *Megathyrsus maximus and Brachiaria brizanta,* which presented similar colonization rates, regardless of cultivar^38–40^.

Table 2 shows the genera identified in the soil of the grasses investigated. The genera Acaulospora and Glomus were identified in all plants investigated, while the genus Slerocystis was identified to be associated only with *B. brizantha piatã*.

**Table 2:** Presence (1) and absence (0) of genera of arbuscular mycorrhizal fungi associated with the rhizosphere of *different forage grasses* in Cerrado soil.

Forage	*Acaulospora*	*Claroideglomus*	*Diversispora*	*Scutellospora*	*Sclerocystis*	*Glomus*	*Gigaspora*
*B. decumbens*	**1**	**1**	**1**	**1**	**0**	**1**	**1**
*B. Ruziziensis*	**1**	**1**	**1**	**0**	**0**	**1**	**1**
*B. Brizantha Marandu*	**1**	**1**	**1**	**1**	**0**	**1**	**1**
*B. Brizantha Piatan*	**1**	**1**	**1**	**0**	**1**	**1**	**1**
*B. brizantha cv. Xaraes*	**1**	**0**	**1**	**1**	**0**	**1**	**1**
*B. brizantha cv. Paiaguas*	**1**	**1**	**0**	**1**	**0**	**1**	**1**
*B. brizantha cv. Ipyporan*	**1**	**0**	**1**	**1**	**0**	**1**	**1**
*B. brizantha cv. Humidicola*	**1**	**0**	**1**	**0**	**0**	**1**	**1**
*P. maximum cv. Mombasa*	**1**	**0**	**0**	**0**	**0**	**1**	**1**
*P. maximum cv. Kenya*	**1**	**1**	**1**	**0**	**0**	**1**	**0**
*P. maximum cv. Zuri*	**1**	**1**	**0**	**1**	**0**	**1**	**0**
*P. maximum cv. Aruana*	**1**	**1**	**1**	**1**	**0**	**1**	**0**
*P. maximum cv. Tamani*	**1**	**1**	**1**	**1**	**0**	**1**	**1**
*P. maximum cv. Massai*	**1**	**1**	**1**	**0**	**0**	**1**	**0**

**Figure 2:**
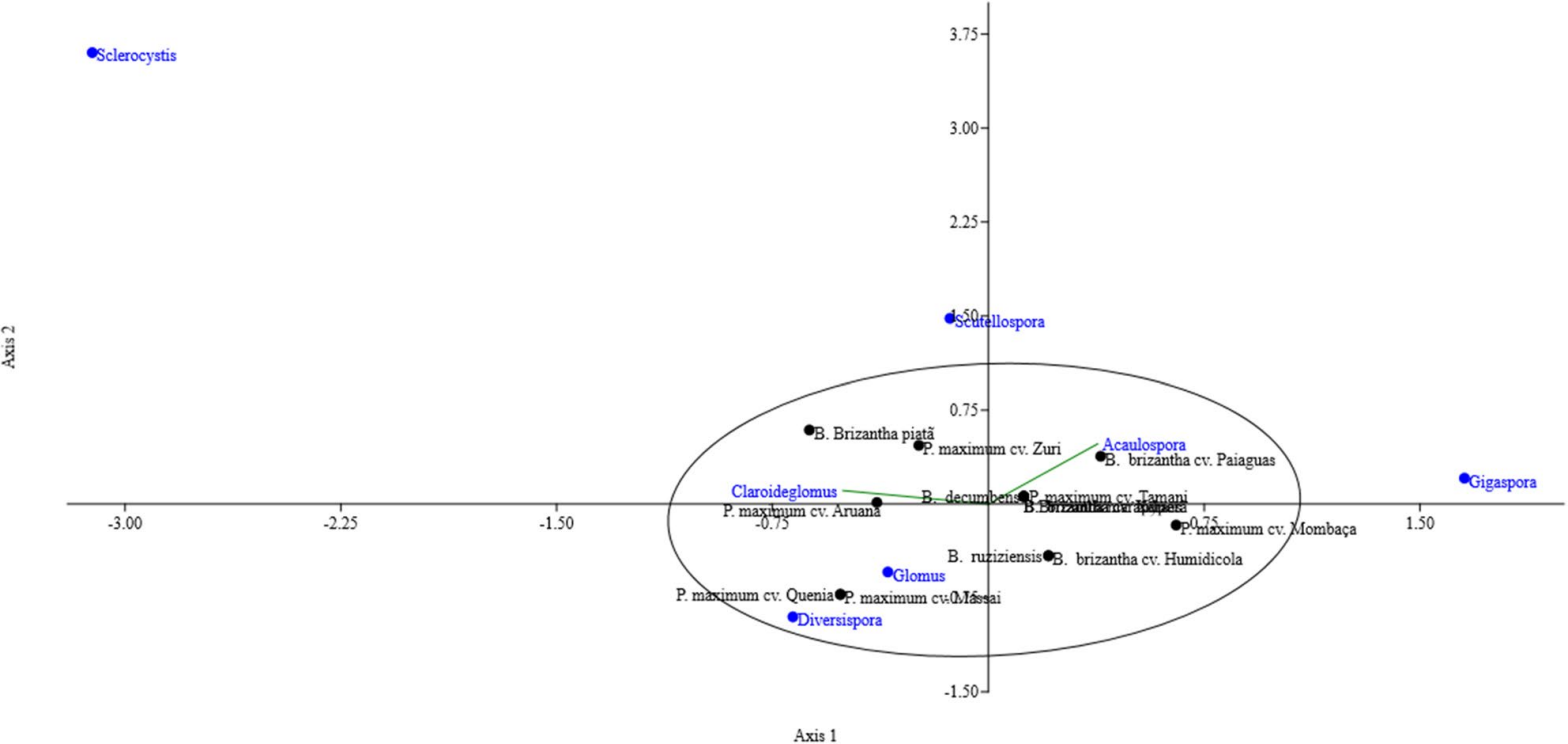
Canonical correspondence analysis of the associated genera found in rhizospheric soil of different *forage grasses* in cerrado soil.

Canonical correspondence analysis aims to identify the proximity of the presence of mycorrhizal fungi identified with the grass species investigated (Fig. 2).

The genera of mycorrhizal fungi identified were commonly found in the rhizosphere of all grasses investigated, except for the genera Gigaspora, Scutelospora and Sclerocysts, which indicates that the association with these genera of fungi is less recurrent than with the other genera. The genera Glomus and Acaulospora are commonly found in Cerrado soils^6^. When investigating the biodiversity of AMF in Cerrado soils, the same genera were found to be associated with bamboo^41^, sugarcane^42,43^, sorghum and corn^44^.

The grasses present substantial mycorrhizal colonization volume for the root system of the grasses, which favors fungal colonization. The adaptability of this plant family to the natural conditions of the Cerrado also favors the exposure of the plant to the action of the fungus when subjected to situations of environmental stress, especially water.

## Conclusion

The spore density values do not vary among the species of fodder studied. This parameter is independent of the plant, as it is a physiological response of the fungus. On the other hand, *Urochloa decumbens* presented higher values of mycorrhizal colonization. The genera of mycorrhizal fungi identified are commonly found in the rhizosphere of all grasses investigated, except for the genera *Gigaspora, Scutelospora and Sclerocysts*, and these genera of fungi are less recurrent than the other genera.
