# Author Correction: Mycorrhizal fungi arbuscular in forage grasses cultivated in Cerrado soil

**DOI:** 10.1038/s41598-022-07665-8

**Published:** 2022-03-01

**Authors:** Leidiane dos Santos Lucas, Aurelio Rubio Neto, Jadson Belem de Moura, Rodrigo Fernandes de Souza, Maria Eduarda Fernandes Santos, Lorena Fernandes de Moura, Elitania Gomes Xavier, José Mateus dos Santos, Ryan Nehring, Sandro Dutra e Silva

**Affiliations:** 1 Graduate Studies in Agricultural Sciences / Agronomy, Instituto Federal Goiano, Rio Verde, Goiás Brazil; 2 Sedmo - Soil Research Group, Ecology and Dynamics of Organic Matter, Evangelical College of Goianésia, Goianesia, Goiás Brazil; 3 Graduate Studies in Natural Resources of the Cerrado, State University of Goiás, Anápolis, Goiás Brazil; 4 SEMPA Seeds Technology, Goiania, GO Brazil; 5 Graduate Studies in Social, Technological and Environment Science, Evangelical University of Goiás, Anápolis, Goiás Brazil; 6 Department of History and Philosophy of Science, University of Cambridge, Cambridge, UK

Correction to: *Scientific Reports* 10.1038/s41598-022-07088-5, published online 24 February 2022

The Acknowledgments section in the original version of this Article was incomplete.

“This research was funded in whole, or in part, by the Wellcome Trust [Grant number 217968/Z/19/Z]. For the purpose of open access, the author has applied a CC BY public copyright license to any Author Accepted Manuscript version arising from this submission.”

now reads:

“Acknowledgments to the Foundation for Research Support in the State of Goiás - FAPEG, National Council for Scientific and Technological Development - CNPq, Evangelical Educational Association - AEE and Federal Institute of Goiás - IFGoiano. This research was funded in whole, or in part, by the Wellcome Trust [Grant number 217968/Z/19/Z]. For the purpose of open access, the author has applied a CC BY public copyright license to any Author Accepted Manuscript version arising from this submission.”

The original Article has been corrected.

